# Educational Gradients Behind Medical Adverse Event Deaths in the US—A Time Series Analysis of Nationwide Mortality Data 2010–2019

**DOI:** 10.3389/fpubh.2022.797379

**Published:** 2022-06-15

**Authors:** Petteri Oura

**Affiliations:** ^1^Department of Forensic Medicine, Faculty of Medicine, University of Helsinki, Helsinki, Finland; ^2^Forensic Medicine Unit, Finnish Institute for Health and Welfare, Helsinki, Finland; ^3^Center for Life Course Health Research, Faculty of Medicine, University of Oulu, Oulu, Finland

**Keywords:** education, socioeconomic status, adverse event, mortality, US

## Abstract

**Background:**

Deaths due to medical care appear common. Individuals with low socioeconomic position seem to be at a higher risk for sustaining a medical adverse event and premature death. This time series analysis aimed to assess educational gradients behind adverse event deaths in the US over the period 2010–2019.

**Methods:**

Publicly available mortality and census data were retrieved from official sources. The data included age, sex, educational attainment, and underlying cause of death. Adverse event deaths were identified by ICD-10 codes Y40—Y84 and Y88. Four education categories were created in accordance with the International Standard Classification of Education 2011 coding scheme [No high school or General Educational Development (GED); High school or GED; Some college; Bachelor's degeree or higher]. To capture also highly educated individuals, the analysis was delimited to ≥30-year-olds. Age-adjusted mortality rates (AMRs) were compared between education categories by means of mortality plots and linear mixed models.

**Results:**

A total of 25,897,334 certified deaths occurred among ≥30-year-olds during the study period. The underlying cause of death was an adverse event in a rarity of cases (0.12%, *n* = 31,997). Individuals with Bachelor's degeree or higher had the lowest adverse event AMRs (6.1–12.4 per million per year), followed by the Some college category (9.6–18.6), the High school or GED category (17.1–35.4), and finally the No high school or GED category (20.0–36.0). AMRs showed a gradual increase as education level decreased (*p* ≤ 0.001 against those with Bachelor's degeree or higher). Moreover, the temporal increase in adverse event AMRs was more pronounced among individuals with low than high education; the contrasts between categories were greatest toward the end of the study period.

**Conclusion:**

The findings of this study suggest that the widening socioeconomic gradients in mortality extend also to fatal adverse events. Future studies should aim to analyze whether access to care, severity of the condition at presentation, quality of care, and social determinants of health may drive the gradients.

## Introduction

Unintended injuries caused by medical management are commonly referred to as medical adverse events (1, 2). In this study, the term is used to capture a wide range of cases, both avoidable or unavoidable, including late complications and sequelae of injuries caused by medical management. Even though deaths due to medical care appear common (3–5), the theme has been lacking scientific attention (3, 6). Further efforts should be directed to the research and prevention of medical adverse events (7–13).

Socioeconomic gradients in illness and death are potentially avoidable and may thus be influenced to increase population health (14, 15). In particular, the quantification of socioeconomic gradients behind mortality is considered a crucial public health priority globally (16). Of the wide range of indicators available, education has been recommended as the general proxy for socioeconomic status, as it is considered a sufficiently reliable measure which remains relatively stable throughout adulthood. Importantly, it may be harmonized across countries to enable comparisons at the international level, and is considered more robust against reverse causality than other socioeconomic proxies such as income level (14, 17, 18).

Evidence has accumulated that low socioeconomic position is associated with premature mortality (19–21). Although individuals with low socioeconomic position seem to be at a higher risk for medical adverse events (22–24), previous research on socioeconomic gradients behind adverse event deaths has been scarce. Building on the official US mortality data from 2010 to 2019, this nationwide time series analysis aimed to characterize educational gradients behind medical adverse event deaths among ≥30-year-old individuals. Given the previous evidence on the association between low socioeconomic position, adverse events and premature all-cause mortality, low education level was hypothesized to be associated with higher adverse event mortality.

## Materials and Methods

### Dataset

The material of this study stemmed from US governmental organizations. The source population were US residents whose mortality data were retrieved from National Center for Health Statistics (Centers for Disease Control and Prevention, Department of Health and Human Services) and population data from Census Bureau (Department of Commerce) and Bureau of Labor Statistics (Department of Labor). The analysis was delimited to individuals aged ≥30 years in order to secure individuals from all education levels, including those at bachelor-level or higher.

Publicly available databases, all cleared of identifiable information, were queried in April 2022, conforming to the regulations set by the organizations. All queries were made strictly for research purposes. Ethical approvals were not sought as the analysis was fully registry-based and utilized already public datasets.

### Mortality

Mortality data were obtained from the National Vital Statistics Online Data Portal, where “Mortality Multiple Cause” packages were retrieved from the years 2010–2019. The data included the decedents' age (accuracy of 1 year), sex (male/female), education (please see Section Education Level below), and underlying cause of death. Causes of death were documented in accordance with the 10th revision of the International Classification of Diseases coding system (ICD-10) (25).

Adverse event deaths, including late complications and sequelae of adverse events, were proxied by ICD-10 codes Y40—Y84 and Y88. These included the following subcategories (6):

- Complications of medical or surgical procedures without misadventure (Y83—Y84 and Y88.3)
- Medical or surgical misadventures (Y60—Y69 and Y88.1)
- Medication-related adverse events (Y40—Y59 and Y88.0)
- Device-related adverse events (Y70—Y82 and Y88.2).

In this study, complications of procedures were referred to as “procedure-related events.” Misadventures, medication-related events, and device-related events were combined as “other adverse events” as they were rare.

The annual numbers of all adverse event deaths, prodecure-related deaths, and other adverse event deaths were obtained in a piecewise manner, documenting each separately for age (10-year intervals), sex (males/females), and education categories (please see Section Education Level below).

### Population

Population estimates were obtained using the Current Population Survey (CPS) Explore Data tool, where “CPS Basic Monthly” packages were retrieved for the years 2010–2019. The January population count was used as the at-risk population of the respective year. Population counts were obtained in a piecewise manner, separately for 10-year age intervals, sex (males/females), and education category (please see Section Education Level below). The 2010 population distribution was used as the reference for age-adjusted mortality rates.

### Education Level

Table 1 presents the categories used to document educational attainment in the original mortality and census data. In mortality datasets, education was reported according to either the 1989 or 2003 revision of the death certificate; a key for combining the two versions has been proposed previously (26).

**Table 1:** Definitions of education categories and accordance with original data sources.

**Education category used in this study**	**Accordance with original data source**		
	**Mortality data, 1989 revision**	**Mortality data, 2003 revision**	**Census data**
No high school or GED	No formal education; 1–8 years of elementary school; 1 year of high school; 2 years of high school; 3 years of high school	8th grade or less; 9–12th grade, no diploma	<1st grade; 1st, 2nd, 3rd or 4th grade; 5th or 6th grade; 7th or 8th grade; 9th grade; 10th grade; 11th grade; 12th grade, no diploma
High school or GED	4 years of high school	High school graduate or GED tests completed	High school graduate
Some college	1 year of college; 2 years of college; 3 years of college	Some college credit, but no degree; Associate degree	Some college, but no degree; Associate degree, occupational/vocational; Associate degree, academic program
Bachelor's degree or higher	4 years of college; 5 or more years of college	Bachelor's degree; Master's degree; Doctorate or professional degree	Bachelor's degree; Master's degree; Professional school degree; Doctorate degree

*GED, General Educational Development*.

Conforming to the International Standard Classification of Education (ISCED) 2011 scheme (27), education data were converted to four mutually exclusive categories, as shown in Table 1.

### Statistical Analysis

The general characteristics of the decedents were presented using percentages and frequencies for categorical variables, and medians and interquartile ranges (IQRs) for continuous variables.

Crude mortality rates (per million per year) were calculated as follows: number of deaths over a year/population count at the start of the year x 1,000,000. Crude mortality rates were calculcated in a piecewise manner for each age, sex, and education category. Then, age-adjusted mortality rates (AMRs) were calculated using the 2010 population distribution as the reference. Educational gradients in adverse event AMRs were illustrated by mortality plots.

Educational gradients were further quantified by constructing two-level linear mixed models with adverse event AMR as the outcome, and education category, year, and education^*^year interaction as predictors. Annual data were considered to be nested within education categories. Year was mean-centered to allow interpretation relative to a 1-year change over time. Sex^*^education and sex^*^education^*^year interactions were also explored but these were not statistically significant; as such, sex stratification was not deemed necessary. A complete-case approach was justified as only ≤ 4.4% of the decedents had missing data on education history. Beta coefficients, 95% confidence intervals (CI) and *P*-values were documented. Betas were interpreted as follows: Intercept indicates AMR in the reference education category (i.e., Bachelor's or higher) at the middle of the study period. Year indicates change in AMR relative to a 1-year change over time. Education indicates average difference in AMR between the reference category (i.e., Bachelor's or higher) and the remaining categories. Education^*^year indicates average difference in AMR relative to a 1-year change over time between the reference category (i.e., Bachelor's or higher) and the remaining categories.

Stata/MP version 17 (StataCorp, College Station, TX) and IBM SPSS Statistics version 27 (IBM, Armonk, NY) were used to perform the statistical analyses. Microsoft Excel version 2005 (Redmond, WA) was used to generate mortality plots. The threshold for statistical significance was set at *P* = 0.05.

## Results

A total of 25,897,334 certified deaths occurred among ≥30-year-olds during the study period. The underlying cause of death was an adverse event in a rarity of cases (0.12%, *n* = 31,997); procedure-related events accounted for the majority of adverse events. General characteristics of decedents in terms of age, sex, and education are presented in Table 2.

**Table 2:** General characteristics of the decedents. The dataset comprised ≥30-year-old decedents.

	**2010**	**2011**	**2012**	**2013**	**2014**	**2015**	**2016**	**2017**	**2018**	**2019**
Total deaths^a^	2,387,791	2,435,211	2,463,782	2,518,291	2,547,373	2,630,126	2,656,674	2,727,093	2,757,141	2,773,852
All adverse events^b^	0.10 (2,365)	0.10 (2,473)	0.10 (2,497)	0.10 (2,644)	0.10 (2,451)	0.10 (2,567)	0.12 (3,082)	0.16 (4,301)	0.16 (4,467)	0.19 (5,150)
Procedure-related events^b^	0.08 (1,847)	0.08 (1,980)	0.08 (2,007)	0.09 (2,180)	0.08 (1,996)	0.08 (2,074)	0.10 (2,589)	0.14 (3,751)	0.14 (3,896)	0.16 (4,572)
Other adverse events^b^	0.02 (518)	0.02 (493)	0.02 (490)	0.02 (464)	0.02 (455)	0.02 (493)	0.02 (493)	0.02 (550)	0.02 (571)	0.02 (578)
**Age of decedents**										
Median (IQR)	78 (65–87)	78 (65–87)	78 (65–87)	78 (65–87)	78 (65–87)	78 (65–87)	77 (65–87)	77 (65–87)	77 (65–87)	77 (65–87)
*Missing*^a^	0	0	0	0	0	0	0	0	0	0
**Sex of decedents**										
Male^b^	49.4 (1,179,229)	49.4 (1,201,888)	49.6 (1,221,217)	49.8 (1,254,117)	50.1 (1,275,989)	50.1 (1,318,599)	50.5 (1,341,661)	50.6 (1,381,131)	50.9 (1,403,687)	51.2 (1,419,681)
Female^b^	50.6 (1,208,562)	50.6 (1,233,323)	50.4 (1,242,565)	50.2 (1,264,174)	49.9 (1,271,384)	49.9 (1,311,527)	49.5 (1,315,013)	49.4 (1,345,962)	49.1 (1,353,454)	48.8 (1,354,171)
*Missing*^a^	0	0	0	0	0	0	0	0	0	0
**Education of decedents**										
No high school or GED^b^	24.6 (588,013)	23.9 (581,825)	23.2 (570,866)	22.5 (565,669)	21.7 (551,680)	20.8 (546,260)	20.5 (543,854)	19.7 (537,873)	19.0 (525,159)	18.5 (512,127)
High school or GED^b^	42.2 (1,008,017)	42.4 (1,031,958)	42.5 (1,046,046)	42.6 (1,071,984)	42.6 (1,086,108)	41.3 (1,086,384)	42.7 (1,134,600)	42.8 (1,166,165)	42.9 (1,182,627)	42.9 (1,189,317)
Some college^b^	16.3 (388,666)	16.6 (405,135)	17.1 (420,331)	17.4 (438,329)	17.9 (455,339)	17.8 (467,458)	18.5 (490,884)	18.8 (512,729)	19.0 (523,044)	19.2 (533,347)
Bachelor's degree or higher^b^	14.7 (349,893)	14.9 (362,903)	15.3 (376,753)	15.6 (393,637)	15.9 (405,358)	15.8 (414,330)	16.6 (440,065)	17.0 (463,514)	17.3 (478,299)	17.7 (490,281)
*Missing*^b^	2.2 (53,202)	2.2 (53,390)	2.0 (49,786)	1.9 (48,672)	1.9 (48,888)	4.4 (114,694)	1.8 (47,271)	1.7 (46,812)	1.7 (48,012)	1.8 (48,780)

^a^*Number of cases. ^b^Percentage relative to total deaths (number of cases). GED, General Educational Development; IQR, Interquartile range*.

**Figure 1:**
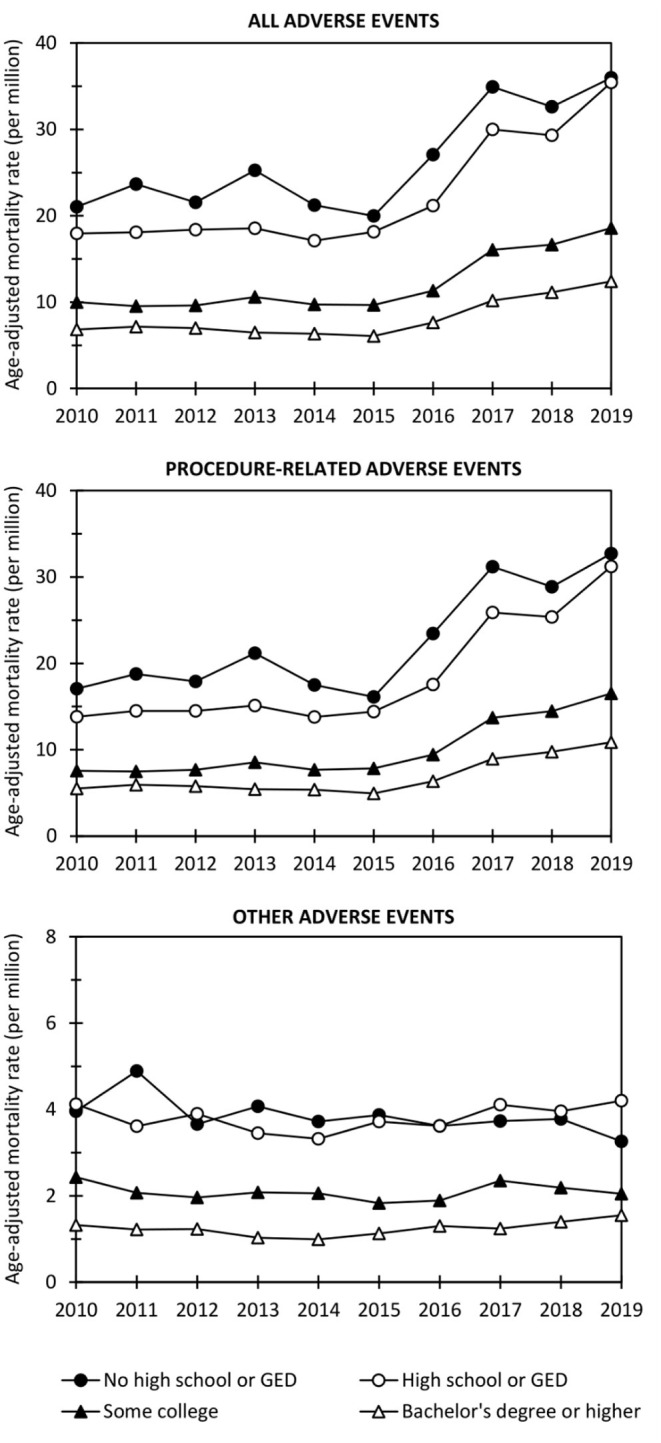
Age-adjusted mortality rates for all adverse events, procedure-related events, and other adverse events in education categories over the period 2010–2019. GED, General Educational Development.

Figure 1 illustrates adverse event AMRs in education categories across the study period. In general, individuals with Bachelor's degeree or higher had the lowest AMR (6.1–12.4 per million per year for all adverse events), followed by the Some college category (9.6–18.6), the High school or GED category (17.1–35.4), and finally the No high school or GED category (20.0–36.0). Procedure-related events mainly drove the trend, whereas other adverse events were rare. The AMRs showed an increasing trend over time, particularly in the No high school or GED and High school or GED categories.

Table 3 presents linear mixed models quantifying the associations of year, education, and education^*^year interaction with adverse event AMRs. Interpreting the year variable, AMRs for all adverse events and procedure-related events generally increased over the study period (*p* ≤ 0.042). Based on the education^*^year interaction, this temporal increase was pronounced among individuals in the No high school or GED and High school or GED categories (*p* ≤ 0.011). Most importantly, however, the models showed strong educational gradients in adverse event mortality; AMRs for all adverse events, procedure-related events, and other adverse events gradually increased as education level decreased. For example, against individuals with Bachelor's degree or higher, the remaining education categories had 4–18 units higher AMRs for all adverse events (*p* ≤ 0.001).

**Table 3:** Two-level linear mixed models with adverse event AMR (per million per year) as outcome, and education, year and education*year interaction as predictors.

	**All adverse events**		**Procedure-related adverse events**		**Other adverse events**	
	**Beta^**a**^ (95% CI)**	***P*-value**	**Beta^**a**^ (95% CI)**	***P*-value**	**Beta^**a**^ (95% CI)**	***P*-value**
Intercept	**8.13 (6.50; 9.76)**	**<0.001**	**6.89 (5.34; 8.44)**	**<0.001**	**1.24 (1.09; 1.39)**	**<0.001**
Year	**0.59 (0.02; 1.16)**	**0.042**	**0.56 (0.02; 1.10)**	**0.041**	0.03 (-0.02; 0.08)	0.307
**Education**						
Bachelor's or higher	Reference		Reference		Reference	
Some college	**4.05 (1.74; 6.36)**	**0.001**	**3.20 (1.01; 5.40)**	**0.004**	**0.85 (0.64; 1.06)**	**<0.001**
High school or GED	**14.29 (11.98; 16.59)**	**<0.001**	**11.73 (9.53; 13.92)**	**<0.001**	**2.56 (2.36; 2.77)**	**<0.001**
No high school or GED	**18.20 (15.90; 20.51)**	**<0.001**	**15.59 (13.39; 17.78)**	**<0.001**	**2.61 (2.41; 2.82)**	**<0.001**
Education*Year						
Bachelor's or higher	Reference		Reference		Reference
Some college	0.39 (−0.42; 1.19)	0.344	0.42 (−0.34; 1.19)	0.279	−0.03 (−0.11; 0.04)	0.345
High school or GED	**1.25 (0.44; 2.05)**	**0.002**	**1.24 (0.48; 2.01)**	**0.001**	0.01 (−0.07; 0.08)	0.888
No high school or GED	**1.04 (0.23; 1.84)**	**0.011**	**1.15 (0.39; 1.92)**	**0.003**	**−0.11 (−0.19;** **−0.04)**	**0.001**

^a^Statistically significant associations are bolded. Betas are interpreted as follows: Intercept indicates AMR in the reference education category (i.e., Bachelor's or higher) at the middle of the study period. Year indicates change in AMR relative to a 1-year change over time. Education indicates average difference in AMR between the reference category (i.e., Bachelor's or higher) and the remaining categories. Education*year indicates average difference in AMR relative to a 1-year change over time between the reference category (i.e., Bachelor's or higher) and the remaining categories. AMR, Age-adjusted mortality rate; CI, Confidence interval; GED, General Educational Development.

## Discussion

This time series analysis of 25,897,334 certified deaths among ≥30-year-old US residents aimed to assess educational gradients behind medical adverse event deaths over the period 2010–2019. In accordance with the a priori hypothesis, lower education was indeed associated with higher adverse event mortality; mortality gradients gradually increased as education level decreased. Moreover, the temporal increase in adverse event mortality was more pronounced among individuals with low than high education.

The quantification of socioeconomic gradients in mortality is a crucial public health priority (16, 28). Previous literature has documented the relationship between lower socioeconomic position and higher mortality using a variety of indicators including education (14, 19–21). Furthermore, previous evidence has suggested a higher risk of adverse events among patients with low socioeconomic status (22–24). The present findings are well in harmony with these data, clearly demonstrating that socioeconomic gradients in mortality extend to fatal adverse events. Interestingly, mortality gradients showed a gradual increase across the four education categories, suggesting a relatively linear relationship between educational attainment up to age 30 years and adverse event mortality.

The present findings support the existence of universal, cross-cutting gradients in adverse event mortality. Speculative explanations can be offered. As previously proposed in the context of surgical procedures (29), the socioeconomic gradients in health outcomes are likely driven by disparities in access to care, severity of the condition at presentation, quality of the medical care itself, and social determinants of health. Since the selection of health care providers and procedures that are available to individuals differ markedly by socioeconomic position, the association with adverse event mortality may be mediated by the number and type of healthcare contacts. As a speculative example, individuals with higher socioeconomic status have easier access to care and may also have higher rates of non-essential procedures such as cosmetic surgery, thus being susceptible to adverse events. On the other hand, patients of disadvantaged origin generally access care at lower quality providers (30) and appear to have higher rates of operative mortality in a variety of procedures (31). As such, future studies are invited to reassess the direction and strength of the gradients after accounting for healthcare contacts.

An alarming finding were the widening disparities between education categories over the study period. In particular, the increase in adverse event mortality was more pronounced among individuals at high-school level or lower, when compared to those at college-level or higher. The contrasts between education categories were greatest toward the end of the study period, implying that the issue is current and requires further exploration. While the present dataset is inconclusive in terms of identifying underlying factors, it can be speculated that the widening mortality gradients are probably attributed to growing disparities in the domains already introduced above, i.e., access to care, severity of the condition at presentation, quality of care, and social determinants of health (29). For example, it has been suggested that access to care may have become more important for the early detection and complication-free treatment of conditions, or that management of conditions may have become more complex and advanced in ways that favor individuals with high socioeconomic status (32). In the big picture, the widening socioeconomic gradients have also concerned all-cause mortality in the US (33); the present data show that adverse event deaths clearly contribute to this disparity. In contrast to the US, however, socioeconomic disparities in all-cause mortality have remained stable or followed a narrowing trend in Canada and Europe. European and Canadian trends in adverse events deaths remain to be studied, and if similar differences between countries are to be confirmed, transnational comparisons may help identify the underlying factors.

The present analysis demonstrated widening educational gradients behind fatal medical adverse events among US residents over the course of a decade, which has not been addressed in previous studies. However, as macro-level data are not sufficient for guiding preventive and counteractive measures, future studies are warranted. Primarily, studies should aim to analyze whether healthcare contacts (e.g., access to care, number and type of healthcare contacts, and quality of healthcare professionals and clinics) have a role in the equation. As suggested by the theory of proportionate universalism, counteractive actions need to be universal but in reasonable proportion to the level of disparity (34, 35). Although contrasts between the two education extremities were highest, it will not suffice to focus on the most disadvantaged groups.

Official data sources, nationwide coverage, and a decade-long study period constitute the main strengths of the study. The data are publicly accessible, enabling confirmatory and subsequent analyses around the topic. Education has been recommended as the most accurate proxy of socioeconomic position and was coded according to ISCED suggestions. However, this study also had limitations. It is generally evident that medical adverse events are underrepresented in cause-of-death statistics (3, 6). The retrospective nature of the data and lack of information on other domains than basic demographic traits prevented more detailed analyses of mediating and confounding factors.

## Conclusion

The quantification of socioeconomic gradients in mortality remains a crucial public health priority. This time series analysis of 25,897,334 certified deaths among ≥30-year-old US residents demonstrated widening educational gradients behind fatal medical adverse events over the period 2010–2019, which has not been addressed in previous studies. Lower education was associated with higher adverse event mortality as mortality gradients gradually increased along with decreasing education level. Moreover, the temporal increase in adverse event mortality was more pronounced among individuals with low than high education; the contrasts between categories were greatest toward the end of the study period. The present findings support the existence of universal, cross-cutting gradients in adverse event mortality. As the present macro-level data are not sufficient for guiding preventive and counteractive measures, future studies are warranted. Speculatively, access to care, severity of the condition at presentation, quality of care, and social determinants of health may drive the gradients.

## Data Availability Statement

Publicly available datasets were analyzed in this study. This data can be found here: https://www.cdc.gov/nchs/data_access/vitalstatsonline.htm#Mortality_Multiple and https://data.census.gov/mdat/#/.

## Ethics Statement

Ethical review and approval was not required for the study on human participants in accordance with the local legislation and institutional requirements. Written informed consent for participation was not required for this study in accordance with the national legislation and the institutional requirements.

## Author Contributions

PO is the sole author of the manuscript. He worked on the conception of the project, data collection, analysis and interpretation of the data and writing the manuscript.

## Conflict of Interest

The author declares that the research was conducted in the absence of any commercial or financial relationships that could be construed as a potential conflict of interest.

## Publisher's Note

All claims expressed in this article are solely those of the authors and do not necessarily represent those of their affiliated organizations, or those of the publisher, the editors and the reviewers. Any product that may be evaluated in this article, or claim that may be made by its manufacturer, is not guaranteed or endorsed by the publisher.

## Abbreviations

AMR: age-adjusted mortality rate
CI: confidence Interval
CPS: Current Population Survey
GED: General Educational Development
ICD-10, International Classification of Diseases: 10th revision
IQR: interquartile range
ISCED: International Standard Classification of Education
P: *P*-value
US: United States of America.
